# Primary breast lymphoma initially diagnosed as invasive ductal carcinoma: A case report

**DOI:** 10.1002/ccr3.4189

**Published:** 2021-05-05

**Authors:** Natsuki Uenaka, Shinya Yamamoto, Seiya Sato, Takamichi Kudo, Shoko Adachi, Kazutaka Narui, Mikiko Tanabe, Akimitsu Yamada, Takashi Ishikawa, Itaru Endo

**Affiliations:** ^1^ Department of Breast and Thyroid Surgery Yokohama City University Medical Center Yokohama Japan; ^2^ Department of Breast Surgery and Oncology Tokyo Medical University Shinjuku‐ku Japan; ^3^ Department of Pathology Yokohama City University Medical Center Yokohama Japan; ^4^ Department of Gastroenterological Surgery Yokohama City University Graduate School of Medicine Yokohama Japan

**Keywords:** breast, immunohistochemical staining, lymphoma

## Abstract

A malignant tumor in the breast may not be conclusive of breast cancer. It is important to keep the possibility of primary breast lymphoma in rare scenarios. For the diagnosis of primary breast lymphoma, immunohistochemical staining is necessary.

## Graphical abstract

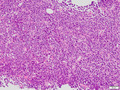

## 1 INTRODUCTION

A 73‐year‐old woman presented with a right breast mass. Radiological findings were consistent with breast cancer. Pathological analysis of the core needle biopsy specimen indicated triple‐negative invasive ductal carcinoma. However, additional immunohistochemical studies indicated primary breast diffuse large B‐cell lymphoma, which is difficult to diagnose by imaging and pathological findings.

Primary breast lymphoma (PBL) is very rare, accounting for 0.04%‐0.5% of all malignant breast tumors.^1^ PBL has the following characteristics: (a) a tumor in the breast, (b) no history of lymphoma, (c) lymphoma has a close association with the breast tissue in the pathological specimen, and (d) no lymphadenopathy except for in the ipsilateral axillary lymph node.^2^ Most PBLs are diffuse large B‐cell lymphoma (DLBCL), which accounts for approximately 40%‐70% of all PBL cases.^3^

We report a case of DLBCL in the breast initially treated as breast cancer.

## 2 CASE HISTORY/EXAMINATION

A 73‐year‐old woman visited the hospital with complaints of a painless palpable mass more than 1‐month duration in her right breast. She was diagnosed with a malignant lesion after fine‐needle aspiration, and she was transferred to our hospital. She had no B symptoms (unexplained weight loss, fever, or night sweats). She had no family history of breast cancer.

**FIGURE 1:**
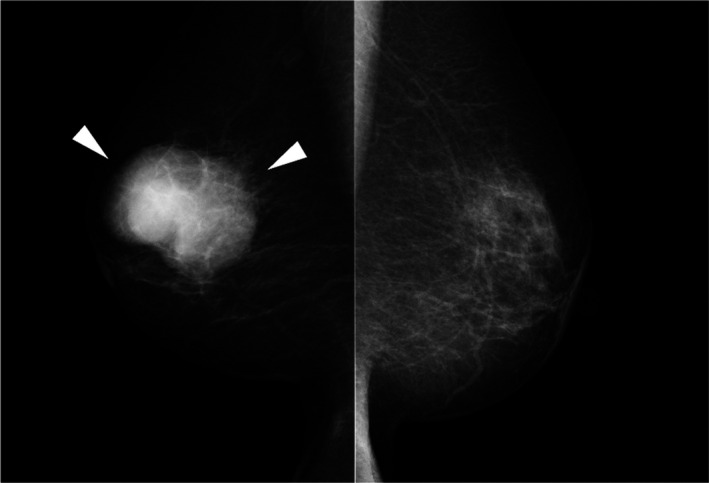
Mammography in the mediolateral oblique position. A high‐density mass with microlobulated margins in the middle area (arrowhead)

**FIGURE 2:**
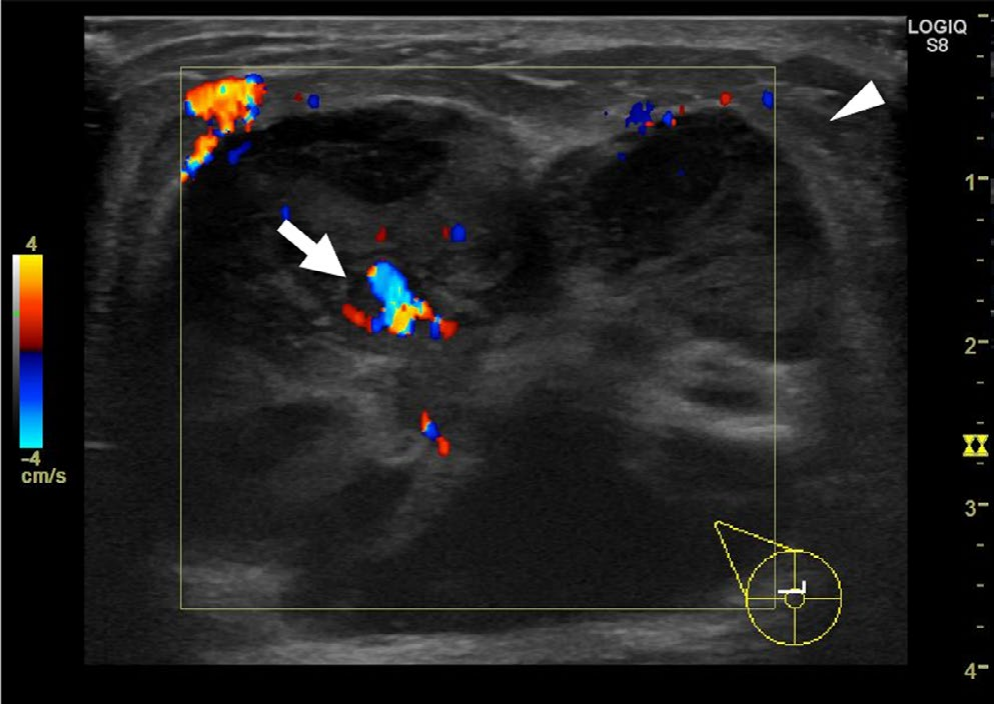
Ultrasonography. A 47‐mm irregular hypoechoic mass with heterogeneous internal echo (arrowhead) and blood flow in the mass (arrow)

**FIGURE 3:**
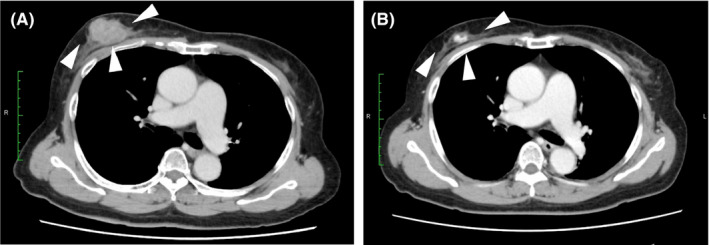
Contrast‐enhanced computed tomography (CT). A, CT before chemotherapy showing the mass in the right breast (arrowhead). B, CT after chemotherapy showing remarkable mass shrinkage (arrowhead)

**FIGURE 4:**
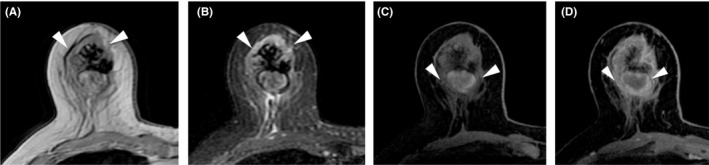
Magnetic resonance imaging. A, T1‐weighted images. The edge of mass is isointense (arrowhead). B, T2‐weighted images. The edge of mass is hyperintense (arrowhead). C, Contrast‐enhanced T1‐weighted images in the early phase (arrowhead). D, Contrast‐enhanced T1‐weighted images in the late phase (arrowhead)

Physical examination showed a hard mass in the upper to lower outer quadrant of the right breast measuring 50 mm, and there were no palpable axillary lymph nodes. Mammography showed a high‐density mass with microlobulated margins (Figure 1). Ultrasonography revealed a 47‐mm irregular hypoechoic mass with a heterogeneous internal echo and blood flow (Figure 2). Contrast‐enhanced computed tomography showed a mass in the right breast and no metastatic lesions (Figure 3A). Magnetic resonance imaging (MRI) revealed that the edge of the mass was isointense on T1‐weighted imaging (Figure 4A) and hyperintense on T2‐weighted imaging (Figure 4B). The time‐intensity curve of contrast‐enhanced T1‐weighed imaging showed a slow plateau pattern (Figure 4C and D).

Levels of all tumor markers, including carcinoembryonic antigen and carbohydrate antigen 15‐3, were within the normal ranges. Her lactate dehydrogenase level was 207 IU/L.

**FIGURE 5:**
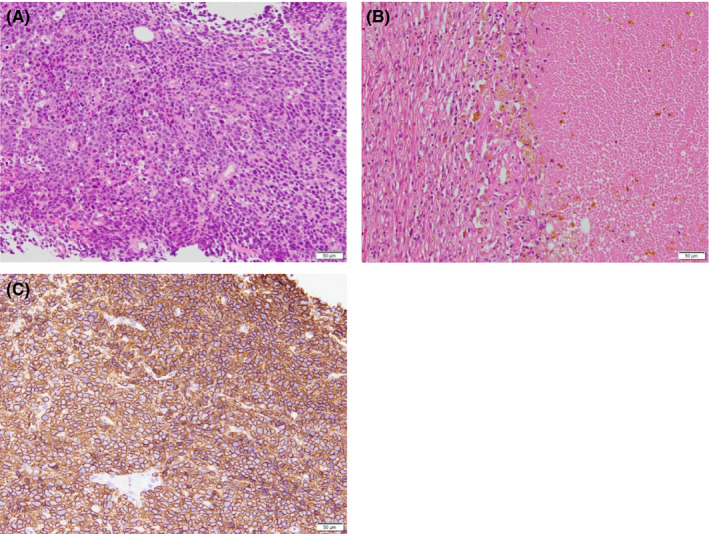
Pathological and immunohistochemical analysis. A, Hematoxylin‐eosin staining of the core needle biopsy specimen indicating solid proliferation of atypical cells with rounded nuclei (×400). B, Hematoxylin‐eosin staining of the surgical specimen indicating no residual tumor (×400). C, The resected specimen is positive for CD20 (×400)

Analysis of a core needle biopsy (CNB) specimen revealed solid proliferation of atypical cells with rounded nuclei (Figure 5A). Therefore, the lesion was identified as invasive ductal carcinoma, solid type, nuclear grade 3 (nuclear atypia: 3, mitotic count: 3). Immunohistochemical (IHC) staining showed that the tumor was negative for estrogen receptor, progesterone receptor, and human epidermal growth factor 2. The Ki67 labeling index was 80%.

## 3 DIFFERENTIAL DIAGNOSIS, INVESTIGATIONS, AND TREATMENT

A diagnosis of triple‐negative breast cancer (cT2N0M0) was made based on the pathological and imaging findings. Because the patient had atrial fibrillation, anthracycline‐containing regimens were avoided. Therefore, 12 weeks of neoadjuvant weekly paclitaxel (80 mg/m^2^) were scheduled. However, due to cerebral infarction, chemotherapy was ended after eight cycles. The mass had shrunk remarkably after only eight cycles (Figure 3B), so she underwent total mastectomy and sentinel lymph node biopsy.

Pathological examination of the resected specimen revealed no tumor cells; hence, we made a diagnosis of a pathological complete response (pCR) (Figure 5B). However, a pathologist reexamined the CNB specimen, and a diagnosis of PBL was considered. IHC staining indicated positivity for leukocyte common antigen (LCA), CD20 (Figure 5C), Bcl‐6, and MUM‐1, with borderline positivity for CD10. Therefore, the diagnosis was corrected to DLBCL, nongerminal center type. The Ann Arbor stage was 1E because there was one extralympatic site.

## 4 OUTCOME AND FOLLOW‐UP

The patient received R‐CHOP (rituximab, cyclophosphamide, doxorubicin, vincristine, and prednisone) chemotherapy based on the suggestion of a hematologist. She was scheduled to receive six cycles, but R‐CHOP administration was terminated after five cycles due to hematologic toxicity requiring blood transfusion. No recurrence has been observed 2 years after chemotherapy.

## 5 DISCUSSION

We experienced a case of PBL initially treated as invasive ductal carcinoma. PBL is difficult to diagnose based on both imaging and pathological findings.

There are few imaging features that distinguish PBL and breast cancer. However, PBL shows characteristic features on MRI.^2^ PBL is hyperintense on T2‐weighted images and isointense on T1‐weighted images. With regard to time‐intensity curves, most PBLs display type 2 kinetic curves (slow or rapid enhancement and plateau in the delayed phase). Type 1 (enhancing pattern) and type 3 (rapid enhancement and washout in the delayed phase) kinetic curves are less common. In our case, the MRI findings were consistent with the diagnosis of PBL. However, as 33.6% of breast cancers also have type 2 kinetic curves,^4^ it can be difficult to differentiate PBL from breast cancer based solely on MRI findings.

It is also difficult to distinguish PBL and breast cancer based solely on pathological findings.^5^ In a study by Lin et al^6^ 7 of 41 (17%) hematologic malignant lesions in the breast were misdiagnosed as breast cancer. PBL and breast cancer cells are often morphologically similar; therefore, it may be difficult to distinguish them by hematoxylin‐eosin staining alone. DLBCL, which was the histological type in our case, is characterized by a diffuse proliferation of large lymphocytes with large nuclei.^7^ These features can mimic invasive breast cancer, solid type, or invasive lobular carcinoma. When diffuse proliferation of cells is observed in breast tumors, the epithelial or hematologic origin should be confirmed by IHC staining, especially if the tumor shows other findings, such as hormone receptor negativity, no intraductal lesions, and a high N/C ratio, that do not actively support breast cancer. Guilbert et al reported a similar proposal.^8^ Yoneyama et al reported a similar case, and they highlighted several characteristics, including conspicuous apoptosis, marked nuclear rays due to crushing, and polymorphism in tumor cells, that distinguish breast cancer from PBL.^5^ Lin et al also identified several characteristics as findings suspicious of lymphoma, such as lack of in situ lesions, frequent individual karyorrhectic cells, lymphoepithelial lesions, and cellular discohesiveness.^6^ On the other hand, on hematoxylin and eosin staining, the shape of the cells, the lack of similar glandular shape, the uniform appearance of the tumor tissue, and the lack of invasiveness into the mammary ducts may have been findings that would have raised suspicion of lymphoma.

A malignant tumor in the breast may not be conclusive of breast cancer. Other possible malignancies in the breast area, such as lymphoma, should be considered as a differential diagnosis by clinicians and pathologists. In cases where the findings are not typical of breast cancer, examining a larger tissue sample using biopsy may help make an accurate diagnosis.

Immunohistochemical staining is useful to confirm that a breast lesion is lymphoma and is required to determine the histological type. LCA is a specific leukocyte marker used to identify lymphoid neoplasms.^9^ CD20 is used to determine whether a neoplasm originated from B cells.^7^ Other B‐cell markers include CD19, CD22, CD79, and CD45.^7^ DLBCL is classified into two types, namely germinal center B cell–like type and nongerminal center B cell–like type, depending on the expression patterns of CD10, Bcl‐6, and MUM‐1.^10^

Because of the low frequency of PBL, there is no standard treatment. Chemotherapy for PBL is generally administered based on the recommendation for systemic lymphoma of the same histological type.^11^ Our patient achieved pCR after 8 weeks of weekly paclitaxel. The standard treatment for DLBCL is R‐CHOP, and paclitaxel‐based regimens are rarely administered. Rizzieri et al reported the effectiveness of weekly paclitaxel for recurrent or refractory aggressive non‐Hodgkin lymphoma. In their study, 26% of patients achieved pCR.^12^ Casasnovas et al also reported a Phase II study of paclitaxel in patients with refractory and relapsed aggressive non‐Hodgkin lymphoma, in which 4.4% of patients achieved CR.^13^ Therefore, the response to paclitaxel need not indicate that the tumor was not DLBCL.

According to previous reports, surgery does not improve the prognosis in PBL,^5^, ^14^ and surgery is recommended only for diagnostic purposes. Therefore, in our case, surgery was deemed to be unnecessary. A proper initial diagnosis is important to avoid overtreatment. In addition, administering paclitaxel as neoadjuvant chemotherapy might have also been an overtreatment. Although the patients achieved pCR while taking paclitaxel, additional chemotherapeutic agents were still administered.

Sozzi et al^15^ reported a multicenter analysis on PBL. In their study, the 5‐year overall survival, disease‐free survival, and local control rates were 53%, 41%, and 87%, respectively. They concluded that local control is good, but systemic recurrence occurs frequently. No recurrence has been observed within 2 years after chemotherapy in our presented case; however, a longer follow‐up is necessary.

In conclusion, we reported a case of PBL initially treated as breast cancer. It can be difficult to distinguish between primary breast lymphoma and breast cancer based on pathological findings. For the accurate diagnosis of PBL, IHC staining is necessary, particularly when the tumor is hormone receptor‐negative, has no intraductal lesions and no high N/C ratio. A malignant tumor in the breast may not be conclusive of breast cancer. It is important for both clinicians and pathologists to keep the possibility of PBL in rare scenarios.

## CONFLICT OF INTEREST

None declared.

## AUTHOR CONTRIBUTIONS

NU: acquired the data, analyzed, and interpreted the data; wrote the manuscript; approved the final manuscript. SY: analyzed and interpreted the data; wrote the manuscript; approved the final manuscript. MT: wrote the manuscript; approved the final manuscript. SS, TK, SA, KN, AY, TI, and IE: analyzed and interpreted the data; approved the final manuscript.

## Data Availability

Data sharing not applicable to this article as no datasets were generated or analyzed during the current study.
